# Associations of pre-pregnancy body mass index and gestational weight gain with pregnancy outcome and postpartum weight retention: a prospective observational cohort study

**DOI:** 10.1186/1471-2393-14-201

**Published:** 2014-06-11

**Authors:** Margaretha Haugen, Anne Lise Brantsæter, Anna Winkvist, Lauren Lissner, Jan Alexander, Bente Oftedal, Per Magnus, Helle Margrete Meltzer

**Affiliations:** 1 Division of Environmental Medicine, Norwegian Institute of Public Health, P.O. Box 4404, Nydalen NO-0403 Oslo, Norway; 2 Department of Internal Medicine and Clinical Nutrition, Sahlgrenska Academy, University of Gothenburg, Gothenburg, Sweden; 3 Department of Public Health and Community Medicine, Public Health Epidemiology Unit, Sahlgrenska Academy, University of Gothenburg, Gothenburg, Sweden; 4 Division of Epidemiology, Norwegian Institute of Public Health, P.O. Box 4404, Nydalen NO-0403 Oslo, Norway

**Keywords:** MoBa, Body mass index, Prepregnant, Gestational weight gain, Birth outcome, Postpartum weight gain

## Abstract

**Background:**

Excessive gestational weight gain (GWG) is associated with pregnancy complications, and Norwegian Health Authorities have adopted the GWG recommendations of the US Institute of Medicine and National Research Council (IOM). The aim of this study was to evaluate if a GWG outside the IOM recommendation in a Norwegian population is associated with increased risk of pregnancy complications like hypertension, low and high birth weight, preeclampsia, emergency caesarean delivery, and maternal post-partum weight retention (PPWR) at 6 and 18 months.

**Methods:**

This study was performed in 56 101 pregnant women included in the prospective national Norwegian Mother and Child Cohort Study (MoBa) in the years 1999 to 2008. Women who delivered a singleton live born child during gestational week 37 to 42 were included. Maternal prepregnant and postpartum weight was collected from questionnaires at 17^th^ week of gestation and 6 and 18 months postpartum.

**Results:**

A weight gain less than the IOM recommendations (GWG < IOM rec.) increased the risk for giving birth to a low weight baby among normal weight nulliparous women. A weight gain higher than the IOM recommendations (GWG > IOM rec.) significantly increased the risk of pregnancy hypertension, a high birth weight baby, preeclampsia and emergency cesarean delivery in both nulliparous and parous normal weight women. Similar results were found for overweight women except for no increased risk for gestational hypertension in parous women with GWG > IOM rec. Seventy-four percent of the overweight nulliparous women and 66% of the obese women had a GWG > IOM rec. A GWG > IOM rec. resulted in increased risk of PPWR > 2 kg in all weight classes, but most women attained their prepregnant weight class by 18 months post-partum.

**Conclusions:**

For prepregnant normal weight and overweight women a GWG > IOM rec. increased the risk for unfavorable birth outcomes in both nulliparous and parous women. A GWG > IOM rec. increased the risk of a PPWR > 2 kg at 18 months in all weight classes. This large study supports the Norwegian Health authorities’ recommendations for normal weight and overweight women to comply with the IOM rec.

## Background

The global obesity epidemic affecting women of reproductive age is a major contributor to adverse pregnancy and birth outcomes [1,2]. Excessive gestational weight gain has been associated with an increase in adverse birth and pregnancy outcomes independent of prepregnancy weight [3,4]. The US Institute of Medicine (IOM) synthesized the state-of-the-art knowledge about pregnancy outcome in relation to prepregnant body mass index (BMI) and gestational weight gain in their recommendations in 2009 [5]. The Norwegian Directorate of Health has adopted these recommendations, which take into account prepregnancy BMI; and while normal weight women are recommended to gain 11.5 -16 kg, obese women are recommended to gain no more than 5–9 kg during pregnancy (Table 1). The purpose of these guidelines is to reduce perinatal morbidity and mortality and to reduce health problems later in life for both mother and child [6,7]. IOM has based their recommendations on an extensive review of the scientific literature, but their conclusions have been questioned by more recent research. One research report based on more than 170,000 deliveries in Germany criticised the guidelines for giving too narrow limits for optimal gestational weight gain (GWG) ranges [8], while other studies requested more detailed information about recommended GWG aimed at the different obesity classes [9,10]. Gestational weight loss has also been suggested for heavily obese women (class II and III), while others did not find weight loss advisable in obese women in class I and II, due to increased risk for prematurity and for small for gestational age (SGA) babies [11,12].

**Table 1:** **The American Institute of Medicine (IOM) recommendations for gestational weight gain **[5]

**Prepregnant BMI categories**	**According to IOM recommendations**
< 18.5	12.5 – 18 kg
18.5-24.9	11.5 – 16 kg
25 – 29.9	7 – 11.5 kg
>30.0	5-9 kg

In a study from 2009 it was pointed out that parous women had lower risk of small babies, i.e. small for gestational age (SGA) at a much lower GWG than nulliparous women, suggesting to reduce the GWG recommendations for parous women [13]. In a more recent study from a US cohort it was confirmed that optimal GWG was related to parity but that the risk of increased postpartum weight retention was increased in parous women compared to nulliparous women [14].

According to the IOM report, excessive GWG has been found to result in increased risk of large for gestational age (LGA) babies independent of prepregnant BMI [4]. Excessive GWG has also been found to be an independent predictor for child obesity while complying with the IOM guidelines resulted in lower frequency of adiposity in the offspring at 6 years of age [15,16].

With use of data from The Norwegian Mother and Child Cohort Study (MoBa), a large prospective pregnancy cohort recruiting pregnant women during the years 1999 to 2008, we wanted to evaluate the risk to be born with low and high birth weight, SGA, LGA, pregnancy hypertension, preeclampsia and emergency caesarean deliveries among nulliparous and parous women with a GWG outside the IOM guidelines in term delivered babies. A second aim was to evaluate the guidelines in relation to postpartum weight retention (PPWR) at 6 and 18 months postpartum.

## Methods

### Population and study design

The Norwegian Mother and Child Cohort Study (MoBa) is a prospective population-based pregnancy cohort study conducted by the Norwegian Institute of Public Health [17]. Participants were recruited from all over Norway from 1999–2008. The women consented to participate in 40.6% of the pregnancies. The cohort now includes 114.500 children, 95.200 mothers and 75.200 fathers. Follow-up is conducted by questionnaires at regular intervals and by linkage to national health registries. This present study uses the quality assured data files made available for research in 2010 (version 5). Informed consent was obtained from each MoBa participant upon recruitment. The study was approved by The Regional Committee for Medical Research Ethics in South-Eastern Norway.

**Figure 1:**
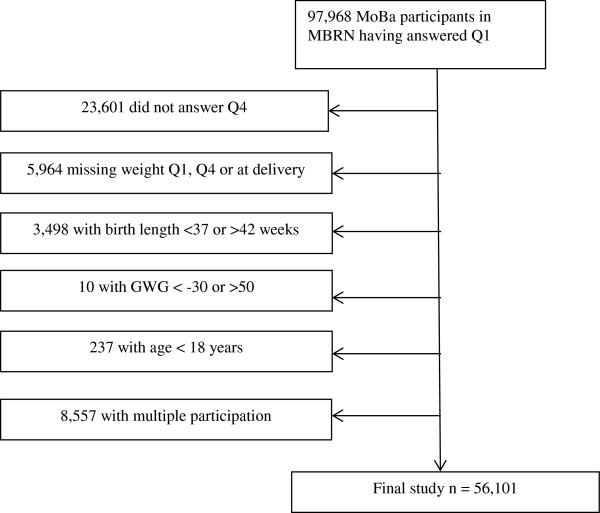
Flow diagram for inclusion of participants for the study from the Norwegian mother and child cohort.

When preparing the dataset, 97 968 women had answered questionnaire 1 (Q1), the baseline MoBa questionnaire covering socio-demographic information and general health and were recorded in The Medical Birth Registry (MBRN) [18] with a singleton delivery. The women had to have completed questionnaire 4 (Q4), a questionnaire answered around 6 months postpartum, excluding further 23 601 women. To be included, prepregnant weight and height (Q1) and weight at delivery and at 6 months postpartum (Q4) had to be recorded (excluding n = 5 964). We excluded participants with a pregnancy duration <37 weeks or >42 weeks (n = 3 498) and if GWG was less than −30 kg or higher than 50 kg (n = 10). Lastly, we excluded women <18 years of age (n = 237) and women with a second or third participation in MoBa (n = 8 557), leaving a study sample of 56 101 women for analysis of health outcomes up to 6 months postpartum (Figure 1). Weight at 18 months was only obtained from 36 606 (65%) of the women and of these 409 were either pregnant again or had had another child, leaving 36 197 women for analyses of health outcomes at 18 months.

### Outcome variables

Birthweight was measured by the midwife who attended the birth and reported in the MBRN. Low birth weight was defined as below 2500 g and high birth weight was defined as above 4500 g at birth. SGA and LGA were calculated as below the 10^th^ percentile and above the 90^th^ percentile of population based birthweight according to gender and week of gestation [19]. Hypertension and preeclampsia occurrence were collected from MBRN where hypertension is given as hypertension during pregnancy without other complications and preeclampsia is defined two visit after 20 weeks of gestation with a systolic blood pressure ≥ 140 or diastolic blood pressure ≥ 90, and two urinary protein of 1+ or greater. Participants with hospital-confirmed eclampsia and hemolysis, elevated liver enzymes, and low platelet count (HELLP-syndrome) were included as valid cases, as well as those with preeclampsia superimposed on chronic hypertension. Preeclampsia diagnosis in MBRN has been validated with a positive occurrence in 82% of the cases [20]. Emergency caesarean deliveries are defined as acute caesareans in MBRN.

PPWR at 6 months and 18 months was calculated from self-reported weight at 6 months (PPWR_6mo) and self-reported weight at 18 months postpartum (PPWR_18mo).

### Exposure variable

GWG in kg was calculated from self-reported weight at delivery and registered 6 months after birth. GWG was divided into three categories according to the IOM definitions; below recommended weight gain (GWG < IOM rec.), according to recommended weight gain (GWG = IOM rec.) and above recommended weight gain (GWG > IOM rec.) for all BMI classes (Table 1).

### Other variables

Prepregnant height and weight were self-reported at week 17 in pregnancy (Q1) and prepregnant BMI was calculated as kg/m^2^. Prepregnant BMI was categorized according to the WHO classification as underweight (<18.5 kg/m^2^), normal weight (18.5-24.9 kg/m^2^), overweight (25.0-29.9 kg/m^2^) and obese (≥30.0 kg/m^2^). From Q1 we also collected data about maternal educational attainment categorized into four categories (≤12, 13–16, ≥17 years and missing), and women were categorized according to their smoking habits in pregnancy as non-smokers, occasional smokers and daily smokers.

Maternal age at delivery was reported in MBRN and gestational age was calculated from expected date of delivery on the basis of first trimester ultrasound. If this ultrasound measure was missing, gestational age was calculated from last menstrual period. Parity was based on data from both MoBa and MBRN and categorized into two categories, nulliparous and parous (0, 1). Maternal diabetic condition were collected from MBRN and categorized into a 0,1 variable including diabetes type I and II as well as gestational diabetes. Smoking postpartum was collected from Q4. Data on breastfeeding practice were gathered from Q4 and used as a continuous variable in months of any breastfeeding or missing data. Information about breastfeeding between 6 and 12 months postpartum was collected from the questionnaire answered at 18 months postpartum and categorized into a 0,1 variable and breastfeeding > 12 months was categorised into another 0,1 variable.

### Statistical methods

All analyses were run on nulli- and parous separately. All values are given as mean (SD) and calculated for each BMI prepregnant category separately. To evaluate the effect of GWG on the birth outcomes we used multiple logistic regression adjusted for potential confounding by maternal age, maternal height, gestational length, maternal smoking in pregnancy, maternal education and diabetic condition. To estimate adjusted odds for SGA and LGA with birthweight within the 10^th^ and 90^th^ percentile as reference, multinomial logistic regression was used (with GWG < IOM rec. and GWG > IOM rec.) with GWG = IOM rec. as reference category. In the same way we evaluated PPWR at 18 months < 0 kg and > 2 kg by multinomial logistic regression. Results are presented as odds ratio (OR) with 95% confidence intervals (CI). In the models calculating the impact of GWG on PPWR, breastfeeding practice and postpartum smoking were adjusted for in addition to maternal age and maternal education. All models were checked for violations from the model assumptions.

In an attempt to evaluate if the IOM recommendations were too narrow for normal weight and overweight women we used the model approach described by Beyerlein et. al [8]. First we performed multiple linear regression with birth weight as the dependent variable and GWG as the explanatory variable and adjusted for: child’s gender, parity, gestational age, maternal age at delivery, maternal height, prepregnant BMI, smoking in pregnancy and diabetic condition. Interaction terms with GWG and all covariates were included to identify possible effect modifiers. Effect size was considered statistically significant for p-values < 0.05. Only the interaction term GWG*BMI came out significant (p < 0.001) while the interaction term GWG* maternal age was borderline significant (p = 0.048). Logistic regression models were run for SGA and LGA separately to evaluate the risk in terms of GWG adjusted for maternal age at delivery, maternal height, smoking in pregnancy, parity and diabetic condition. The SGA and LGA percentiles are gender and gestational age specific and hence these variables were not adjusted for [19]. The prediction models for SGA and LGA were estimated using the estimated logistic regression coefficients and confounders fixed at their means and modes as appropriate [8]. GWG values in the range of −30 to 50 kg were explored. The risk of adverse birth weight outcome was assessed as the sum of the predicted risk of SGA and LGA in prepregnant normal weight and overweight women, leaving underweight and obese women with too little power to be explored. We evaluated the IOM recommendations in these weight classes where a predicted value should have been 20% since SGA is defined as the 10^th^ percentile of the birthweight and LGA as the 90^th^ percentile of the birth weights with regard to gender and gestational length. All statistical analyses were performed using statistical software PASW statistics 20 (SPSS Inc., IBM Company, Chicago, Ill, USA). All p- values <0.05 were considered statistically significant.

## Results

The nulliparous women were younger, had a lower prepregnant BMI, had higher education and smoked less than the parous women (Table 2). The nulliparous women also had a higher GWG, a lower PPWR at 6 months, but significantly higher weight gain at 18 months compared with the parous women.

**Table 2:** Demographic data of nulliparous and parous women (n = 56 101) in the Norwegian mother and child cohort study, 1999-2008

	**Nulliparous**	**Parous**	
	**n = 29 931**	**n = 26 170**	**p-value**
Mean (SD) age at delivery (y)	28.4 (4.3)	31.8 (4.1)	<0.001
Mean (SD) prepregnant BMI (kg/m^2^)	23.7 (4.1)	24.2 (4.2)	<0.001
Prepregnant BMI categories (%)			
< 18.5	3.2	2.5	
18.5-25	68.7	64.1	
25-30	20.0	23.7	<0.001
>30	8.1	9.7	
Class 1	6.0	7.2	
Class II	1.6	1.9	
Class III	0.5	0.6	
Mean (SD) height (m)	1.68 (0.06)	1.68 (0.06)	ns
Education (%)			
High school or less	27.5	35.8	
College 3 years	43.9	41.0	
Masters and higher	26.5	21.2	<0.001
Missing	2.1	2.0	
Smoking in pregnancy (%)			
Not smoking	90.5	88.3	
Occasional smoking	3.0	2.7	
Daily smoking	4.7	6.5	<0.001
Missing	1.8	2.5	
Mean (SD) gestational weight gain (kg)	15.5 (6.1)	14.7 (5.9)	<0.001
Mean (SD) postpartum weight retention at:			
6 months (kg)	1.2 (5.0)	1.3 (4.5)	0.016
18 months (kg)	**n = 19604**^**§**^	**n = 16593**^**§**^	
	2.1 (5.7)	0.8 (5.1)	<0.001

^§^Denotes number of participants available to analyses at 18 months postpartum.

The percentage of babies born with low birth weight was 2.4% in the nulliparous underweight group and 1.2% in the parous underweight group. In the other BMI classes less than 1% was born with low birthweight. The percentage born with high birth weight was 6.5% in nulliparous and 12.3% in parous obese women. The rate of SGA was highest in the underweight group in both nulliparous and parous women (16.5% and 9.4% respectively) and lowest in the overweight nulliparous and obese parous women (6.0% and 2.9% respectively). In the obese group of nulliparous and parous women, 15.0% and 27.1% were born LGA respectively.

For normal weight nulliparous women a GWG < IOM rec. increased the odds for giving birth to a low weight baby (OR = 2.16 (95%C.I. 1.57, 2.96)), while for parous women the odds was borderline statistically significant (OR = 1.56 (95%C.I. 0.99, 2.45)), while no increased odds were seen for the other weight classes. For a GWG > IOM rec. increased odds for getting a baby with high birth weight were found for all weight classes except for the underweight women (Table 3). For gestational hypertension, preeclampsia and emergency caesarean delivery increased odds were found for both nulliparous and parous normal weigh and overweight women. The same was seen for the obese women, but the odds did not reach statistical significance.

**Table 3:** **Adjusted odds ratios**^**† **^**and 95% confidence interval for a gestational weight gain higher than the IOM’s recommendation (GWG > IOM rec.) with gestational weight gain according to the IOM’s recommendations (GWG = IOM rec.) as reference for birth outcomes among 29 931 nulliparous and 26 170 parous women**

**Weight class**	**High birth weight (>4500 g)**	**Gestational hypertension**	**Preeclampsia**	**Emergency caesarean delivery**
**Nulliparous**				
Underweight	Not estimable^§^	Not estimable^§^	1.83 (0.75,4.50)	1.71 (0.85,3.43)
Normal weight	2.65 (2.09,3.35)***	1.76 (1.41,2.20)***	2.44 (2.03,2.92)***	1.44 (1.28,1.62)***
Overweight	1.57 (1.05,2.35)*	1.55 (1.03,2.32)*	2.87 (1.96,4.88)***	1.42 (1.14,1.77)**
Obese	1.50 (0.95,2.37)	1.32 (0.80,2.17)	1.70 (1.17,2.47)**	1.39 (1.04,1.84)*
**Parous**				
Underweight	2.90 (0.70,12.15)	Not estimable^§^	4.63 (0.50,42.93)	1.25 (0.40,3.91)
Normal weight	2.03 (1.73,2.38)***	1.60 (1.14,2.25)**	2.19 (1.62,2.96)*	1.48 (1.23,1.78)***
Overweight	2.04 (1.56,2.66)***	1.14 (0.71,1.83)	1.50 (1.01,2.24)*	1.95 (1.41,2.69)***
Obese	2.19 (1.55,3.11)***	1.54 (0.91,2.59)	1.49 (0.96,2.32)	1.21 (0.85,1.73)

*p < 0.05, **p < 0.01 and ***p < 0.001.

^†^Adjusted for maternal age at delivery, maternal height, maternal education level, smoking in pregnancy, gestational length and diabetic conditions. ^§^ Too few cases.

**Figure 2:**
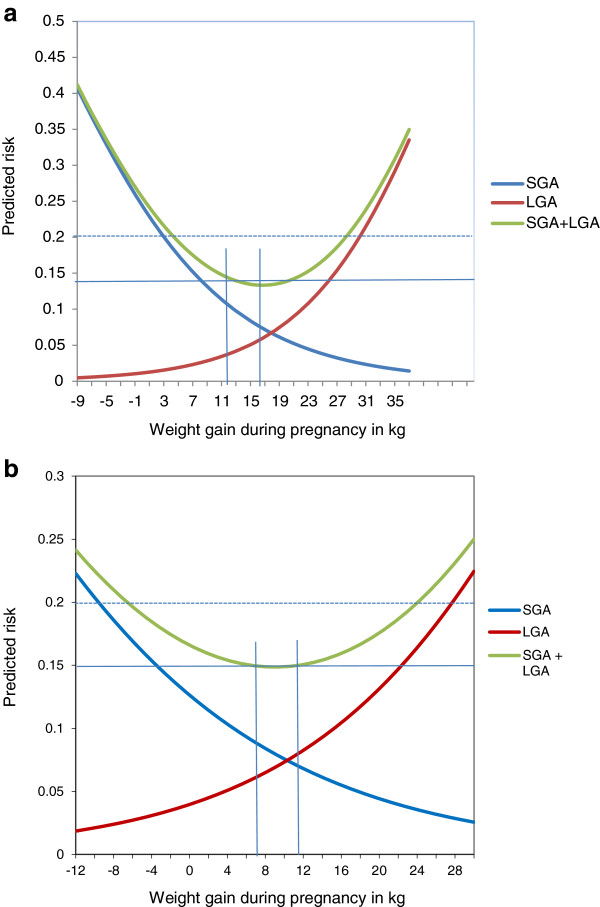
**Predicted optimal gestational weight gain (GWG) based on small for gestational age (SGA) and large for gestational age (LGA) birth outcomes. (a)** Calculated by logistic regression model in 37 332 normal weight women. The vertical lines indicate IOM’s GWG recommendations for normal weight women. **(b)** Calculated by logistic regression model in 12 178 overweight women. The vertical lines indicate IOMs GWG recommendations for overweight women.

In adjusted multinomial analyses with birthweight within the 10th and the 90th percentile and GWG = IOM rec. as references both nulliparous and parous women had increased odds for getting a LGA baby with GWG > IOM rec. and a reduced odds for getting a SGA baby although not significant for the underweight and obese parous women (Table 4). Increased odds for a SGA baby were seen for underweight and normal weight women with a GWG < IOM rec. while a reduced odds were seen for LGA in all weight classes but only statistically significant for the normal weight women (Table 4).With use of the model described in Statistical methods, we projected the best GWG among normal weight and overweight women with regard to combined predicted risk of giving birth to a SGA and a LGA baby (Figure 2a and b). For the normal weight women and 20% combined predictive risk a weight range for GWG would be between 5 and 28 kg and for overweight women −6 and 24 kg. The IOM rec. in these calculations corresponds to a combined effect of 14% for normal weight women and 15% for the overweight women.

**Table 4:** **Adjusted odds ratio for SGA and LGA in 56 101 women and with children within 10**^**th **^**and 90**^**th **^**birth weight as reference category in multinomial logistic regression**

	**Small-for-gestational-age (SGA)**		**Large-for-gestational-age (LGA)**	
	**Nulliparous**	**Parous**	**Nulliparous**	**Parous**
**Underweight women**	**Adj. OR (95% CI)**	**Adj. OR (95% CI)**	**Adj. OR (95% CI)**	**Adj. OR (95% CI)**
GWG = IOM rec.	1.0	1.0	1.0	1.0
GWG < IOM rec.	1.71 (1.16,2.52)**	2.16 (1.18,3.97)**	Not estimable	0.42 (0.12,1.52)
GWG > IOM rec.	0.39 (0.23,0.67)**	0.55 (0.22,1.37)	1.73 (0.79,3.76)	2.33 (1.03,5.20)*
**Normal weight women**				
GWG = IOM rec.	1.0	1.0	1.0	1.0
GWG < IOM rec.	1.53 (1.34,1.72)***	1.41 (1.18,1.67)***	0.56 (0.44,0.70)***	0.58 (0.49,0.67)***
GWG > IOM rec.	0.63 (0.56,0.71)***	0.64 (0.53,0.77)***	2.17 (1.90,2.48)***	1.73 (1.57,1.91)***
**Overweight women**				
GWG = IOM rec.	1.0	1.0	1.0	1.0
GWG < IOM rec.	1.16 (0.80,1.70)	1.20 (0.78,1.85)	0.47 (0.26,0.83)**	0.83 (0.62,1.11)
GWG > IOM rec.	0.62 (0.48,0.80)***	0.60 (0.44,0.83)**	1.69 (1.27,2.08)***	1.72 (1.46,2.04)***
**Obese women**				
GWG = IOM rec.	1.0	1.0	1.0	1.0
GWG < IOM rec.	1.01 (0.64,1.60)	1.39 (0.72,2.64)	0.69 (0.42,1.12)	0.65 (0.48,0.89)*
GWG > IOM rec.	0.60 (0.42,0.86)**	1.01 (0.55,1.84)	1.69 (1.22,2.34)**	1.61 (1.28,2.03)***

Adjusted for maternal age, maternal height, maternal education, gestational length, smoking in pregnancy and diabetic condition.

*p < 0.05, **p < 0.01 and ***p < 0.001.

The mean PPWR at 6 months was positive for underweight and normal weight women and negative for overweight and obese women with a GWG = IOM rec. (Table 5). For a GWG < IOM rec. a negative PPWR were seen for all weight classes except for the underweight, while a GWG > IOM rec. were associated with positive postpartum weight retentions. Among the nulliparous and parous overweight women 74.1% and 68.1% had a GWG > IOM rec. and among the obese women the figures were 66.3% and 56.1% respectively. The frequency of postpartum weight gain of more than 5 kg at 6 months was 14.5% among the nulliparous women and 8.2% among the parous women (p < 0.001). However, no difference between nulliparous and parous women was seen for those going from normal prepregnant BMI to overweight BMI at 6 months, 12.0% and 12.9% respectively. As seen from the adjusted multinomial logistic regression with a PPWG at 18 months between 0 and 2 kg and GWG = IOM rec. as reference a GWG > IOM rec. significantly increased the odds for a PPWR of more than 2 kg (Table 6). A GWG < IOM rec. increased the odds for a negative PPWR at 18 months among the normal weight and obese women.

**Table 5:** Percent in each weight class gaining weight according to the IOM recommendations, and mean gestational weight gain (GWG) and postpartum weight retention (PPWR) at 6 months post partum among 29 931 nulliparous and 26 170 parous women

	**GWG < IOM rec.**			**GWG = IOM rec.**			**GWG > IOM rec.**		
		**GWG**	**PPWR**		**GWG**	**PPWR**		**GWG**	**PPWR**
**BMI category**	**%**	**kg**	**kg**	**%**	**kg**	**kg**	**%**	**kg**	**kg**
**Nulliparous**									
**Total**	17.9	7.9 (3.6)	−1.9 (4.2)	33.2	13.1 (2.4)	0.0 (3.7)	48.8	19.8 (4.8)	3.1 (5.2)
**Underweight**	26.8	9.7 (2.8)	0.1 (2.8)	46.8	15.4 (1.7)	2.0 (3.2)	26.4	23.3 (4.4)	6.2 (5.2)
**Normal weight**	21.0	8.9 (2.4)	−1.4 (3.4)	38.5	14.0 (1.4)	0.3 (3.2)	40.4	20.9 (3.9)	3.4 (4.4)
**Overweight**	7.3	3.4 (3.2)	−4.0 (5.5)	18.6	9.4 (1.3)	−1.7 (4.4)	74.1	18.6 (5.2)	2.7 (5.6)
**Obese**	14.4	0.4 (4.2)	−6.9 (7.0)	19.3	7.2 (1.4)	−3.4 (6.0)	66.3	16.9 (5.8)	1.8 (7.1)
**Parous**									
**Total**	18.4	7.2 4.1)	−1.4 (4.3)	35.4	12.9 (2.6)	0.0 (3.5)	46.2	19.0 (4.6)	2.8 (4.6)
**Underweight**	30.5	9.8 (2.0)	0.8 (2.5)	46.7	15.2 (1.6)	2.1 (2.8)	22.8	22.3 (3.4)	4.9 (4.5)
**Normal weight**	20.7	8.9 (2.3)	−0.5 (3.2)	41.6	14.0 (1.4)	1.1 (3.0)	37.6	20.5 (3.6)	3.4 (3.9)
**Overweight**	9.7	3.3 (3.3)	−3.2 (5.1)	22.2	9.4 (1.4)	−1.0 (4.1)	68.1	17.6 (4.7)	2.4 (4.9)
**Obese**	20.8	0.2 (4.2)	−6.2 (6.3)	23.1	7.1 (1.3)	−2.4 (5.2)	56.1	15.7 (5.2)	1.2 (6.2)

**Table 6:** Adjusted odds ratio for losing weight at 18 months postpartum and for gaining more than 2 kg among 19 604 nulliparous and 16 593 parous women with use of multinomial logistic regression and a post-partum weight gain of 0–2 kg as reference category

	**PPWR18mo < 0 kg**		**PPWR18mo > 2 kg**	
	**Nulliparous**	**Parous**	**Nulliparous**	**Parous**
**Underweight women**	**Adj. OR (95% CI)**	**Adj. OR (95% CI)**	**Adj. OR (95% CI)**	**Adj OR (95% CI)**
GWG = IOM rec.	1.0	1.0	1.0	1.0
GWG < IOM rec.	1.35 (0.84,2.18)	1.46 (0.86,2.49)	0.48 (0.29,0.79)**	0.68 (0.38 1.23)
GWG > IOM rec.	0.92 (0.46,1.85)	0.41 (0.19,0.91)*	3.06 (1.78,5.25)***	2.02 (1.14,3.60)*
**Normal weight women**				
GWG = IOM rec.	1.0	1.0	1.0	1.0
GWG < IOM rec.	1.70 (1.51,1.92)***	1.32 (1.16,1.49)***	0.89 (0.78,1.02)	0.83 (0.71,0.96)*
GWG > IOM rec.	0.79 (0.71,0.87)***	0.68 (0.61,0.75)***	1.79 (1.62,1.98)***	1.52 (1.36,1.70)***
**Overweight women**				
GWG = IOM rec.	1.0	1.0	1.0	1.0
GWG < IOM rec.	1.47 (0.98,2.22)	1.18 (0.82,1.68)	0.86 (0.54,1.36)	0.76 (0.49,1.16)
GWG > IOM rec.	0.70 (0.55,0.88)**	0.67 (0.54,0.83)***	1.66 (1.30,2.12)***	1.54 (1.21,1.97)**
**Obese women**				
GWG = IOM rec.	1.0	1.0	1.0	1.0
GWG < IOM rec.	3.01 (1.67,5.43)***	1.59 (0.99,2.56)	1.29 (0.66,2.51)	1.02 (0.58,1.81)
GWG > IOM rec.	1.34 (0.92,1.95)	1.03 (0.71,1.50)	2.57 (1.73,3.84)***	2.33 (1.53,3.57)***

Adjusted for smoking at 6 months postpartum, maternal education, maternal age, breastfeeding in months for 6 months, breastfeeding up to 12 months, breastfeeding more than 12 months. The Norwegian Mother and Child Cohort Study, 1999–2008.

*p < 0.05, **p < 0.01 and ***p < 0.001.

Most women stayed in the same weight category 18 months postpartum compared to the prepregnant weight class (Table 7). Between 85% and 90% of the normal weight women stayed in the same weight class among those gaining less weight or according to the IOM rec. Migration to another weight class was highest among the underweight women and lowest among the normal weight women. There was a significant migration in all weight classes according to GWG status (p < 0.001, Chi-square test), except among the obese women. GWG seemed to have less impact among the obese women, especially among the parous women.

**Table 7:** BMI categories at 18 months post-partum in relation to prepregnant BMI and gestational weight gain (GWG) according to IOM recommendations in 19 604 nulliparous and 16 593 parous given as percentage (%) of each prepregnant weight class

**Prepregnant BMI categories**	**GWG < IOM rec.**				**GWG = IOM rec**				**GWG > IOM rec.**			
	**Underw.**	**Normal w.**	**Overw.**	**Obese**	**Underw.**	**Normal w.**	**Overw.**	**Obese**	**Underw.**	**Normal w.**	**Overw.**	**Obese**
**Nulliparous**												
Underweight	**65.1**	33.7	1.2	0	**46.7**	53.0	0.3	0	**26.1**	68.8	5.1	0
Normal weight	2.8	**88.1**	9.0	0.1	1.7	**86.1**	12.0	0.2	0.7	**74.4**	23.5	1.4
Overweight	0	22.6	**60.8**	10.7	0.1	21.9	**66.4**	11.6	0	10.9	**67.8**	21.2
Obese	0	0.9	18.9	**80.2**	0	0.6	15.0	**84.3**	0	0.7	13.5	**85.9**
**Parous**												
Underweight	**66.9**	33.1	0	0	**55.2**	44.3	0.5	0	**36.6**	63.4	0	0
Normal weight	1.8	**90.1**	8.0	0.1	1.3	**89.1**	9.4	0.1	0.4	**82.0**	17.0	0.5
Overweight	0	28.8	**69.9**	8.3	0	19.5	**72.0**	8.5	0	14.9	**71.1**	14.0
Obese	0	1.6	18.9	**79.5**	0	0.8	20.3	**78.9**	0	0.8	21.1	**78.0**

The bold text indicates the percentage of women belonging to the same weight class 18 mo. postpartum as before pregnancy.

## Discussion

In this study we found that a GWG < IOM rec. increased the risk of low birth weight babies in normal weight nulliparous women, while GWG > IOM rec. significantly increased the risk of high birth weight babies, LGA, development of hypertension, preeclampsia and emergency cesarean deliveries in both nulliparous and parous normal weight and overweight women. A GWG > IOM rec. resulted in increased risk of PPWR > 2 kg in all weight classes, but most women attained their prepregnant weight class at 18 months independent of GWG.

It is known that underweight women have babies with lower birth weight than normal weight women [21,22], and in a meta- analysis it was confirmed that underweight mothers have increased risk of giving birth to babies with low birth weight and SGA [2]. In a case–control study it was shown that gaining less than IOM rec. increased the risk two-fold for a SGA baby compared to a GWG = IOM rec. [23]. A 2.5 odds was also found in a recent study from US, which is in line with our results [24]. Except for the risk of a SGA baby with a GWG < IOM rec. and an increased risk for a LGA baby with a GWG > IOM rec. among parous women, the IOM rec. seemed of less significance for pregnancy and birth outcomes among the underweight women.

A GWG > IOM’s recommendations among the normal weight women in our cohort increased the risk of having babies with high birth weight and LGA, and furthermore of developing hypertension and preeclampsia or under-going emergency cesarean delivery. Results from a US study analyzing the IOM recommendations in relation to SGA and LGA born babies are in agreement with the results in our study [24]. However, a GWG < IOM rec. increased the risk for a baby with low birth weight in nulliparous, but not in parous women, indicating that a higher GWG could be recommended in nulliparous normal weight women. This is in line with other studies which also indicated that a somewhat higher GWG could be acceptable in pre-pregnant normal weight women without increasing the risk for adverse birth outcomes [8,25]. A joint predicted risk of 20% with regard to SGA and LGA in the large German cohort suggested an optimal GWG of 2–18 kg [8]. Our finding that a weight gain between 5 to 28 kg corresponded to a combined predicted risk of 20% for SGA and LGA appears, however, unrealistic. One explanation might be that in our cohort SGA was only found among 7.0% of the babies born to normal weight women, which again might be explained by fewer smokers and higher education level compared to the general population from where the cut off values for SGA and LGA were computed [26]. The IOM recommendations corresponded to a joint predicted risk of about 14%, but the nadir of the curve was not in the center of the IOM recommendations, again indicating a skewed representation in our cohort. Our result from the multinomial regression, however, indicates that the IOM rec. protects well against SGA and LGA and similar results were seen for both nulliparous and parous women.

Prepregnant overweight and obesity have earlier been shown to increase the risk of LGA and babies born with high birth weight [2]. In MoBa it has been shown that increased GWG increases birthweight in a linear association in all weight classes [22], and in this study we found that women with a GWG > IOM rec. had both a higher risk for high birth weight babies and LGA, which also are in agreement with earlier studies [24,27]. For the overweight women the recommended weight gain did not reduce the risk for getting babies with low birth weight or SGA, and this has also been reported in the study to Simas et al. [24]. In the nulliparous overweight women a GWG > IOM rec. increased the risk of developing hypertension and preeclampsia and elective cesarean delivery and similar results were seen for parous women although not statistically significant for gestational hypertension. We performed the same analyses for prediction of an optimal GWG in relation to SGA and LGA among the overweight women as for the normal weight women and a prediction of 20% gave a GWG of −6 to 24 kg. The deviation from the IOM rec. might again be explained by the fact that in this cohort SGA was found in 4.6% and LGA in 15.9% of the overweight women. A joint prediction of 15% comes very close to the IOM rec. and with the nadir in center. A GWG < IOM rec. did not significantly increase the risk for a SGA baby in the multinomial model, leaving the question of a wider weight limit for an optimal GWG in this weight group.

The picture for the obese women is less clear but a GWG > IOM rec. is consistent with increased risk of a LGA child. Ferraro et al. found also that the IOM guidelines were protective for LGA babies in overweight and obese women and advocated the use of the guidelines in the attempt to beat the obesity epidemic in children [4]. However, we found that the IOM guidelines seemed to be less protective for adverse birth outcomes in obese women examined in this study. The importance of obese women avoiding excessive GWG have also been documented in both earlier and more recent studies [1,9,28] and in grossly overweight women weight reduction has been recommended [12,28]. In obese women no increased risk for SGA among those who gained 0.1-4.9 kg during pregnancy has been found [9,11], and our data did not show increased risk of giving birth to a SGA baby for GWG < IOM rec.. This suggests lower recommendations for optimal GWG than the IOM rec., but weight loss in pregnancy has been shown to result in increased delivery of SGA babies among obese women [14]. Strategies for weight reduction before pregnancy seem to be desirable to reach healthy birth outcomes among obese women, but few studies in overweight and obese women have investigated the effect of weight loss prior to conception on health-related variables in women and children [29].

We found that over 50% of the overweight and obese women had a GWG > IOM re. and similar figures have been found in other studies [24,27,30]. Gaining more than the recommendations was associated with a high risk of postpartum weight gain at 18 months while gaining less only increased the risk for PPW loss among normal weight and obese nulliparous women. Similar result with increased PPWR with a GWG > IOM rec. was found in a study looking at PPWR at 12 months [30]. Among underweight women, between 30 and 70% entered the normal weight category 18 months postpartum, but going from underweight to normal weight might be positive for the general health and cannot be considered an adverse health risk. The finding that as many as 23.5% of normal weight primiparous women entered the overweight group, does not support the arguments for higher GWG recommendations as has been indicated earlier [8,25]. Migration in the obese weight class was similar in all GWG groups which seems odd since we found that there was an increased risk of PPWR > 2 kg with a GWG > IOM rec. compared with a GWG = IOM rec. This may be explained by the fact that a 2 kg increase in body weight does not imply a change in weight category. Looking further into the data showed a negative mean weight change at 18 months postpartum among the parous obese women in all GWG classes (data not shown).

Mean PPWR at 6 months were similar in nulliparous and parous women, which indicate no need for separate GWG recommendations according to parity. However, in other studies parity was found to have an important influence on the risk of having emergency caesarean deliveries, having LGA babies and for PPWR, and a lower GWG in parous compared to nulliparous women was suggested [13,14]. Bodnar et al. tested whether parity, smoking, age, race and height modified the association between GWG and the risk of SGA, LGA, preterm births and unplanned cesarean delivery among normal weight women [31]. Moderate degrees of effects were measured, but to reduce the risk of SGA they found that nulliparous, smoking, black and short women could benefit from a somewhat higher GWG. In a comparable cohort study from Denmark, Nohr et al. investigated parity, smoking in pregnancy, short stature and maternal age as factors which might have influence on an optimal GWG [13]. They found that the only factor of importance was parity, where parous women had a reduced risk of SGA at a lower GWG than the nulliparous. Although not analyzed statistically, our data do not support a higher weight gain for nulliparous compared to parous women. This might be explained by MoBa cohort having few non-Caucasian women included, and that smoking prevalence was low and hence had low impact on birth weight [26].

The strengths of our study include the large and nationwide sample size, linkage to the national birth registry and the detailed information about health, lifestyle and other potential confounding variables. Limitations of this study include the fact that in MoBa the weight change and prepregnant weight class are calculated from self-reported weight. Self-reported weight has been found to underestimate actual measured weight although with a relative small difference among non-pregnant women, but with large errors in a small fraction of participants [32]. After applying a probabilistic bias analysis method Bodnar et al. found that self-reported prepregnant weight attenuated the risk of SGA and LGA in underweight, overweight and severely obese women compared to normal weight women when conventional multivariable logistic regression models were used [33]. That the conventional estimates were biased away from the null might suggests that the associations between self-reported prepregnancy BMI and pregnancy outcomes are slightly overestimated. The relatively low participation rate is another concern in MoBa and women in MoBa are not representative of all pregnant women in Norway. However, an evaluation of differences in prevalence estimates between MoBa participants and a nationally representative pregnant population revealed no statistically significant differences regarding eight evaluated exposure-outcomes [26].

## Conclusion

For prepregnant normal weight and overweight women a GWG > IOM rec. increased the risk for unfavorable birth outcomes in both nulliparous and parous women. A GWG > IOM rec. increased the risk of a PPWR > 2 kg at 18 months in all weight classes. This large study supports the Norwegian Health authorities’ recommendations for normal weight and overweight women to comply with the IOM rec.

## Competing interests

All authors declare that they have no competing interests’ or have any financial competing interests.

## Authors’ contributions

MH, ANLB, HMM, AW and LL formatted the research question and designed the study. MH and BO conducted data collection and analyzed the data. All authors contributed to the drafting, and editing of the manuscript as well as the interpretation of the results. All authors have read and approved the final manuscript.

## Pre-publication history

The pre-publication history for this paper can be accessed here:

http://www.biomedcentral.com/1471-2393/14/201/prepub
